# Pseudoaneurysm of cystic artery stump after laparoscopic cholecystectomy managed successfully with branch hepatic artery embolization using jail technique

**DOI:** 10.1093/jscr/rjae152

**Published:** 2024-03-15

**Authors:** Khizer Masroor Anns, Faheemullah Khan, Muhammad Aman, Jehanzeb Shahid, Tanveer U I Haq, Wasim Ahmad Memon, Muhammad Arif Saeed, Amna Khalid, Khabab Abbasher Hussien Mohamed Ahmed, Saba Akram

**Affiliations:** Medical College, The Aga Khan University, Karachi 74200, Pakistan; Department of Radiology, The Aga Khan University Hospital, Karachi 74200, Pakistan; Department of Radiology, The Aga Khan University Hospital, Karachi 74200, Pakistan; Department of Radiology, The Aga Khan University Hospital, Karachi 74200, Pakistan; Department of Radiology, The Aga Khan University Hospital, Karachi 74200, Pakistan; Department of Radiology, The Aga Khan University Hospital, Karachi 74200, Pakistan; Department of Radiology, James Paget University Hospital, Great Yarmouth NR31 6BN, United Kingdom; Medical College, King Edward Medical University, Lahore, 54000, Pakistan; Faculty of Medicine, University of Khartoum, Khartoum 11111, Sudan; Department of Pathology and Laboratory Medicine, Aga Khan University Hospital, Karachi 74200, Pakistan

**Keywords:** cystic artery, cystic artery stump, hepatic artery, pseudoaneurysm, cholecystectomy, embolization, angioembolization

## Abstract

An unusual manifestation caused by cholecystitis, infection, or iatrogenic damage after cholecystectomy is a pseudoaneurysm of the cystic artery. We report this rare illness in a 64-year-old man who visited the emergency room with hematemesis and anemia. The patient initially experienced acute cholecystitis and then underwent a laparoscopic cholecystectomy following which he developed a cystic artery pseudoaneurysm, secondary to infected fluid collection. Based on the patient’s history and contrast-enhanced computer tomography abdomen, a diagnosis of pseudoaneurysm of the cystic artery was made. Angioembolization of the hepatic artery branch was performed to occlude the pseudoaneurysm.

## Introduction

Cystic artery pseudoaneurysm is a rare presentation that commonly occurs because of inflammation around the gallbladder (GB) or because of the presence of gallstones resulting in cholecystitis [1]. In our case, the patient first developed acute cholecystitis and underwent a laparoscopic cholecystectomy following which he developed a cystic artery pseudoaneurysm, secondary to postoperative collection in the GB fossa. Only about 26%–27% of the cases of pseudoaneurysm developed following a cholecystectomy [2]. We describe a case of a 64-year-old man who presented to the emergency room (ER) with hematemesis and anemia and was diagnosed with cystic artery pseudoaneurysm.

## Case presentation

A 64-year-old man who had hematemesis and anemia arrived at the ER. Upon physical examination, he showed 81-bpm heart rate, 20 breaths per minute respiratory rate, 98% oxygen saturation, and 114/57 mm of Hg blood pressure. His laboratory workup showed a hemoglobin level of 8.5 g/dl (normal 12.3–16.6 g/dl), red blood cell count of 3.67 × 10^12^/L (normal 4.25–6.02 × 10^12^/L), a hematocrit of 27.4% (normal 38.4–50.7%), white blood cell count of 10.9 × 10^9^/L (normal 4.8–11.3 × 10^9^/L), and a platelet count of 427 × 10^9^/L (normal 154–433 × 10^9^/L). After that, one packed cell volume was transfused into him in the ER.

The patient had a history of hypertension, ex-smoking, and repeated episodes of hematemesis containing fresh blood over the previous 3 days. Previously, he was diagnosed with acute cholecystitis and had undergone a laparoscopic cholecystectomy at an outside healthcare facility after which he developed a fever and a wound infection with purulent discharge that persisted for 10 days. Following wound infection, the patient developed an upper gastrointestinal bleed (UGIB) with multiple episodes of hematemesis and melena.

Based on the patient’s worsening condition, an esophagogastroduodenoscopy was performed which revealed an oozing large deep ulcer at the junction of the first and second part of the duodenum (D1 and D2). The patient was administered adrenaline, and the bleeding was stopped. The patient had another episode of UGIB a few weeks after the discharge. He was then admitted to the same facility following which a computer tomography (CT) scan of the patient was ordered.

**Figure 1:**
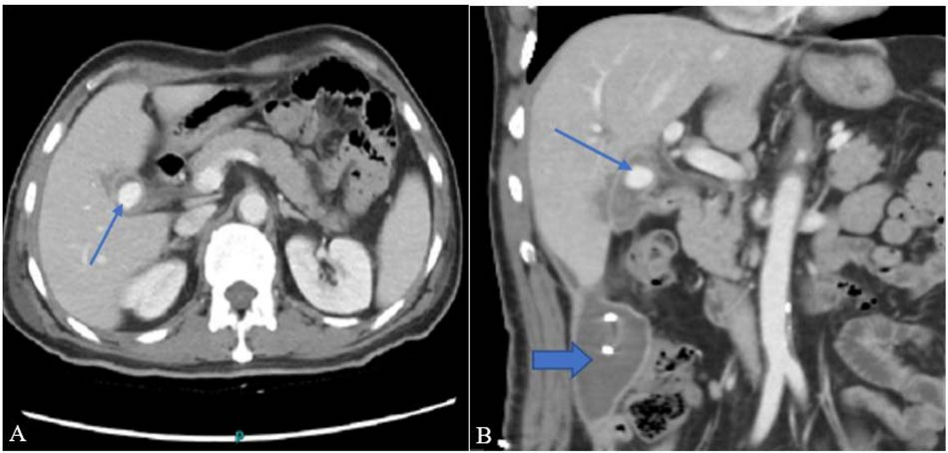
(A) Contrast-enhanced CT axial image through the abdomen shows a small walled-off collection in the GB fossa with a central contrast density representing pseudoaneurysm (arrow). The collection appears to be in communication with the second part of the duodenum. (B) A coronal image from the same CT shows a right para colic gutter collection with an *in situ* pigtail (broad arrow).

Contrast-enhanced computer tomography (CECT) abdomen showed a walled-off collection in the GB fossa measuring 44 × 19 mm with a rent identified in the first segment of the duodenum showing fistulous communication with the collection. There was also another collection, measuring 93 × 54 × 37 mm, in the right paracolic gutter with a thin streak tracing along the subhepatic margin. In addition to the fistula, a post-traumatic focal pseudoaneurysm measuring 13 × 15 mm was seen arising from the right branch of the hepatic artery (Fig. 1A and B). 10F pigtail catheter was placed under CT guidance in the right paracolic gutter collection. Afterwards, the patient was shifted for possible angioembolization to an interventional radiology suit.

**Figure 2:**
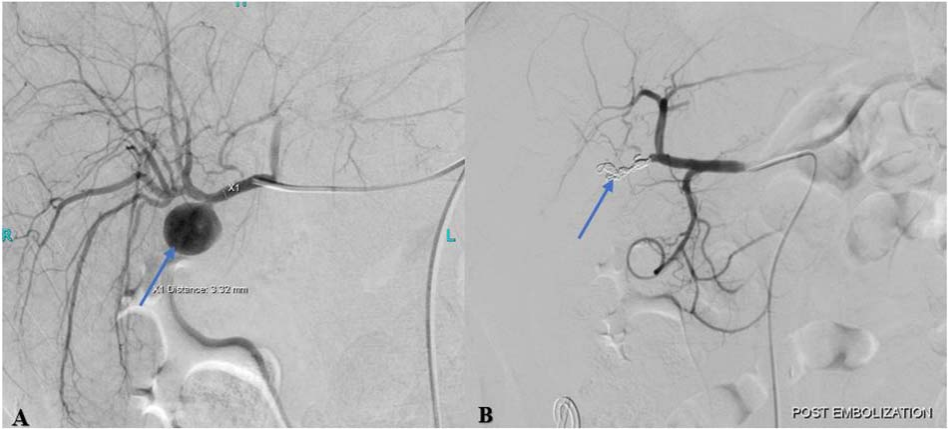
(A) Pre-embolization angiogram of the hepatic artery demonstrates pseudoaneurysm of the cystic artery stump (arrow). (B) Post-coil embolization angiogram of the hepatic artery shows no further filling of the pseudoaneurysm (arrow).

Cannulation of the celiac and superior mesenteric arteries were performed using a 4 French C1 Catheter to perform an angiogram. The angiogram showed a saccular aneurysm measuring 23 × 21 mm arising from the right hepatic artery just at the origin of the cystic artery stump (Fig. 2A). After considering multiple options, it was decided to embolize the hepatic artery proximal and distal to the aneurysm using the jail technique. Selective cannulation of the right hepatic artery was performed with a catheter tip placed distal to the origin of the aneurysm using a Progreat microcatheter. The distal segment was then embolized using three steel coils. The microcatheter was then retracted, proximal to the aneurysm origin followed by proximal segment embolization using three steel coils.

Post-procedural angiogram showed a complete occlusion of the aneurysm and satisfactory filling of the right lobe of the liver through its collaterals from the left hepatic artery (Fig. 2B). Post-procedure hospital stay was unremarkable for any procedure-related complication or hospital-acquired infection. The patient was discharged on the 3rd post-procedure day in stable condition with no output from the abdominal drain.

## Discussion

Pseudoaneurysm, also called a false aneurysm, is a defect of the blood vasculature which involves the out-pouching of the blood vessel because of damage to one or more layers of the vessel wall. A true aneurysm, on the other hand, involves disruption of all three layers of the blood vessel that leads to the expansion of the vessel. Pseudoaneurysms can develop because of several factors that include injury, trauma, infection, or any inflammation of the blood vessel wall [3]. Conditions like hypertension, vasculitis, or atherosclerosis can also damage the vasculature, further weakening the blood vessel wall [4].

Cystic artery pseudoaneurysm is a rare presentation that commonly occurs because of inflammation around the GB or because of the presence of gallstones resulting in cholecystitis [1]. Cases can also occur secondary to any form of iatrogenic injury during a cholecystectomy procedure [3]. Pathogenesis of a pseudoaneurysm because of cholecystitis is not completely known but inflammation and gallstones have been suggestive of the development and rupture of the pseudoaneurysm because of ulceration of the GB wall and erosion of the adventitia of the cystic artery. Moreover, it might as well be caused by the early thrombosis in the cystic artery that develops because of inflammation [3, 5]. Despite the low incidence of vascular injury during cholecystectomy (0.2%–0.5%), the formation of pseudoaneurysm after cholecystectomy may result from any mechanical, i.e. clip application or thermal injury during the procedure. Pseudoaneurysm of the cystic artery may also form because of any biliary spillage, or enteric fistula causing abscess and sepsis. This results in the digestion of the blood vessels [6]. Possible infections, bile leakage or a fistula as mentioned, cause an arterial wall to become fragile and prone to form a pseudoaneurysm [7].

Cholecystitis is found to contribute to 62% of all the reported cases of pseudoaneurysm of the cystic artery, whereas only about 26%–27% of the cases of pseudoaneurysm developed following a cholecystectomy [2]. In our case, the patient first developed acute cholecystitis and underwent an open cholecystectomy following which he developed a pseudoaneurysm of the cystic artery, secondary to an infected collection of fluid in the GB fossa.

Pseudoaneurysm of the cystic artery may rupture and cause hemobilia which occurs because of a hemorrhage into the biliary tract. This is a key differential diagnosis for a pseudoaneurysm in patients who present with an UGIB [8]. Consequently, patients present with a Quincke’s triad, which includes epigastric pain, jaundice, and UGIB [3]. In 86.5% of the patients, they presented with right upper quadrant pain, whereas patients presenting with UGIB manifested classic symptoms of melena and hematemesis, with an endoscopy suggesting a bleed from the Ampulla of Vater [2]. This may lead to pallor and in some cases, a hypovolemic shock requiring a blood transfusion for hemodynamic stability. Moreover, most of the cases of a pseudoaneurysm of cystic artery appear in males, with about 88% of the reported cases being males over the age of 50 [4]. In our case, the patient was a 63-year-old male, who did not have Quincke’s triad but did develop an UGIB with multiple episodes of melena and hematemesis.

Diagnosis of pseudoaneurysm of a cystic artery can be initially made by ultrasound with a color Doppler. A color flow pattern or a cystic mass is suggestive of a pseudoaneurysm, whereas bleeding can be identified as echogenic debris of the intra-vesicular clots. Though safe and inexpensive, ultrasound alone cannot be used for a confirmed diagnosis of a pseudoaneurysm as it is not able to identify the origin of the pseudoaneurysm [4]. A confirmed diagnosis is made by a CT scan, which shows a high-density region associated with the GB that shows an intraluminal bleed. On CECT, a hyperdense lesion is seen around a cystic artery without contrast that enhances the arterial phase [3, 9]. However, hepatic artery angiography is used as the gold standard for diagnosing cystic artery pseudoaneurysm, which was also the case with our patient. This not only helps us identify the pseudoaneurysm directly but serves as an access to embolize the vessel in the management of the pseudoaneurysm. Therefore, it is preferred to perform a hepatic artery angiography initially to decide and plan a proper management plan.

The first line of treatment to manage a patient with a pseudoaneurysm of the cystic artery is a laparoscopic cholecystectomy and the subsequent ligation of the cystic artery [10]. However, a less invasive procedure such as the trans-arterial embolization of the cystic artery is performed using steel coils to occlude the pseudoaneurysm. This is also seen to be effective in patients with a ruptured pseudoaneurysm with unstable hemodynamics [11]. This was also the case with our patient, who was managed with a hepatic artery embolization procedure using the jail technique with coils. This led to a subsequent occlusion of the pseudoaneurysm.

## Data Availability

The data supporting this manuscript's findings is available from the corresponding author upon reasonable request.
